# Are there gender-based variations in the presentation of Autism amongst female and male children?

**DOI:** 10.1007/s10803-022-05552-9

**Published:** 2022-07-13

**Authors:** Sarah Mae Simcoe, John Gilmour, Michelle S. Garnett, Tony Attwood, Caroline Donovan, Adrian B. Kelly

**Affiliations:** 1 School of Applied Psychology, Griffith University, Mt Gravatt, Brisbane, Australia; 2 Institute of Social Science Research, University of Queensland, St Lucia, Brisbane Australia; 3 Attwood and Garnett Events, Brisbane, Australia; 4 School of Psychology and Counselling, Queensland University of Technology, Kelvin Grove, Brisbane, Australia; 5 Centre for Child Health and Well-being, Mental Health and Resilience Theme, Queensland University of Technology, Kelvin Grove, Child Adversity, Brisbane Australia

**Keywords:** Autism, ASD-Level 1, Camouflaging, Gender differences, Gendered behaviour, Social masking, Imagination, Imitation

## Abstract

The Questionnaire for Autism Spectrum Conditions (Q-ASC; Attwood, Garnett & Rynkiewicz, 2011) is one of the few screening instruments that includes items designed to assess female-specific ASD-Level 1 traits. This study examined the ability of a modified version of the Q-ASC (Q-ASC-M; Ormond et al., 2018) to differentiate children with and without ASD-Level 1. Participants included 111 parents of autistic children and 212 parents of neurotypical children (5–12 years). Results suggested that the gendered behaviour, sensory sensitivity, compliant behaviours, imagination, and imitation subscales differentiated autistic females from neurotypical females. Compared to autistic males, autistic females had higher scores on gendered behaviour, sensory sensitivity, social masking, and imitation. Results are discussed in relation to early detection of autistic female children.

In this paper we examine the extent to which autistic female children have characteristics that distinguish them from autistic male children and neurotypical females. This has important implications for early detection and evidence-based service provision for this population. At the outset, we note that in this paper we use *autism-first* language and we use the term *autism* rather than *autism spectrum disorder*. We acknowledge that there are bifurcated views on the use of autism-first language versus person-first language (Autistic Self Advocacy Network, 2021; Bury et al., 2020; Callahan, 2018). We retain the use of autism-first language in recognition of the view that autism is integral to a person’s identity and not an additional aspect, and because this is the preference of advocates and autism experts (Autistic Self Advocacy Network, 2021; Botha et al., 2021; Bury et al., 2020; Department of Social Services, 2021; National Autistic Society, 2021). We understand that views and opinions differ on this point (Botha et al., 2021), but note that the terms ‘autistic person’ and ‘autistic’ are the preferred terms with largest agreement amongst Australian adults who reported having a diagnosis of autism (Bury et al., 2020). We also understand that there are variations in the preferences of autistic people from neurotypical groups (e.g., Callahan, 2018), and we accept that some readers of this paper may disagree with our adoption of autism-first language.

ASD-Level 1 (formally known as Asperger’s syndrome) is a presentation of autism without intellectual or language impairment according to the Diagnostic and Statistical Manual of Mental Disorders – 5th Edition (APA, 2013). ASD-Level 1 is typically diagnosed when individuals demonstrate restrictive/repetitive patterns of behaviours and poor social abilities, but do not display language or cognitive deficits (APA, 2013; Attwood, 2007, 2012). Traditionally, autism has been recognised more frequently in males than females. However, the gender ratio for autism changes with age from 5.5:1 in elementary school children to 2.3:1 for autistic adolescents and between 1.8:1 to 2.57:1 for autistic adults (Posserud et al., 2021; Rutherford et al., 2016). Clinical studies have also suggested that autistic females are more likely to display accompanying intellectual impairment (Mandy et al., 2012), suggesting that females without accompanying intellectual impairment or language delays present differently and are therefore missed or misdiagnosed (Rivet & Matson, 2011; Rynkiewicz & Łucka, 2015). Indeed, it has been found that mental health professionals may reflexively attribute the autistic female presentation to other diagnoses such as Personality Disorders, Schizophrenia, Eating Disorders, Borderline Personality Disorder, Selective Mutism, Separation Anxiety Disorder, Depression, or Specific Phobias (Bulhak-Paterson, 2015; Christensen, 2016; Rynkiewicz & Łucka, 2015). Moreover, common comorbid diagnoses may further complicate accuracy in the assessment protocol for females.

There are several differences in the presentations of autistic males and females, and these differing presentations may be associated with being overlooked in diagnostic interviews (Lai et al., 2015). Relative to autistic males, autistic females have been shown to have an increased ability to camouflage their social confusion (Cook et al., 2021; Goddard et al., 2014; Hull et al., 2019; Simone, 2010; Willey, 2015), and to mimic or imitate genuine reciprocal interactions after carefully evaluating nuances of people’s actions, emotional atmosphere, and social conventions (Attwood, 2007; Bulhak-Paterson, 2015). Compared to autistic males, autistic females have been shown to be more likely to apologise and appease during social situations, which may contrast with their emotional experience (Bulhak-Paterson, 2015; Garnett et al., 2013), and which results in them appearing to have fewer social inadequacies (Bargiela et al., 2016). In their systematic review of empirical literature, Cook et al. (2021) found that autistic camouflaging is associated with significantly poorer mental health outcomes.

There are several differences in the ways in which autistic males and females present when their play with peers is observed, and during a diagnostic assessment. During the elementary school years autistic boys tend to play alone and some distance from peers while autistic girls tend to stay in close proximity to peers and weave in and out of activities, which may camouflage their social challenges to teachers and parents and autistic girls can utilise social opportunities in order to watch the interactions of other girls to mimic social behaviours (Dean et al., 2017). By camouflaging their emotional experience through nuanced imitation, the pervasive and disruptive nature of female social problems remains covert, reducing the likelihood that they will be diagnosed with autism (Bargiela et al., 2016).

The second way that autistic females and males differ in their presentation of autism relates to their special interests. Autistic females may present with special interests that are considered ‘normal’ for their gender and age, such as intense interest in a popular celebrity, television show, or collecting dolls or riding horses (Attwood, 2007; Attwood & Grandin, 2006; Kopp & Gillberg, 2011). Autistic females have also been shown to differ from autistic males in the intensity as well as the focus of their more socially aligned special interests, and may display an intense interest in learning about the conventions of friendship (Attwood, 2012; Supekar & Menon, 2015). Importantly, it has been shown that the choice and pursuit of ‘special interests’ in autistic females appear to be interlaced with camouflage and imitating behaviour, suggesting greater difficulty in identifying unusual ‘special interests’, as required in Section B of DSM-5 (APA, 2013), which may contribute to higher rates of autism diagnosis in males compared to females (McLennan et al., 1993; Rynkiewicz & Łucka, 2015).

The third way that autistic males and females may differ with respect to their presentation relates to gender typicality. Autistic females may not present as gender-typical or may actively reject social gender conventions (Faherty, 2006). Expression of this characteristic may include specific fashion and clothing choices; rejection of fashion trends, and being a ‘tomboy’, or ‘gender-rebel’ (Attwood, 2007; Kopp & Gillberg, 2011). Although anecdotal accounts and clinical observations have noted a higher level of gender incongruence (e.g., gender dysphoria, atypical gender identity) for autistic individuals (Attwood, 2007, 2012; Glidden et al., 2016), research is yet to systematically investigate the co-occurrence of gender identity issues and autism (Glidden et al., 2016; Wood & Halder, 2014). Wood and Halder’s (2014) systematic review found that autistic females displayed more masculine traits than nonautistic (neurotypical) females, which may contribute to clinical observations of gender incongruence among autistic individuals.

As male populations have largely informed diagnostic protocols, unique characteristics of autistic females have been overlooked in research and clinical settings (Glidden et al., 2016). Also, while there are a range of instruments for measuring autism symptomatology, many of these instruments were not developed using female samples, so it is therefore unsurprising that unique characteristics of autistic females have been overlooked.

The most standardised and empirically supported diagnostic assessment used to identify behavioural characteristics and assist in the diagnosis of ASD-Level 1 is the Autism Diagnostic Observation Schedule - Revised (ADOS-2; DiLavore et al., 1995). Methodologically, the ADOS-2 was not adequately standardised on females, nor does it accommodate or conceptualise the proposed social masking and imitation components of the female presentation of autism (Cheslack-Postava & Jordan-Young, 2012; Kamp-Becker et al., 2018; Lai et al., 2015; Rynkiewicz et al., 2016). Similarly, research samples comprising the normative data for the ADOS-2 did not include individuals with the milder characteristics of ASD-Level 1 (Lai et al., 2015). Thus, the ADOS does not allow clinicians and diagnosticians to sufficiently assess the broader range of presentations outside classic autism traits and is yet to adequately inform diagnosis for autistic females (Bargiela et al., 2016; Rivet & Matson, 2011). Indeed, it has been found that adolescent females are at higher risk of misdiagnosis from the ADOS-2 procedure, in spite of their clinical presentation and developmental history suggesting autism (Rynkiewicz & Łucka, 2015).

A recent review of general screening tools for detecting autism in females noted that sex differences may not be captured on standard screening tools (Lundstrom et al., 2019). There are two instruments developed to assess for the features of autism more commonly seen in females. The first is the Autism Spectrum Screening Questionnaire (ASSQ; Ehlers, Gillberg & Wing, 1999) and later versions *(*ASSQ-REV; Kopp & Gillberg, 2011); ASSQ-GIRLS; Kopp & Gillberg, 2011). ASSQ-GIRLS has an 18-item subscale designed to measure characteristics of autism in females (Kopp & Gillberg, 2011). The second is the Questionnaire for Autism Spectrum Conditions (Q-ASC; Attwood et al., 2011). The Q-ASC is a 61-item screening questionnaire completed by parents/caregivers that includes items measuring characteristics of the female presentation. A recent factor analytic study (Ormond et al., 2018) found 8 subscales derived from 31 items, including gendered behaviour, sensory sensitivity, compliant behaviour, friendships and play, social masking, imagination, and imitation (one subscale, talents and interests, was subsequently dropped because of low reliability; Field, 2014; Ormond et al., 2018).

The present study investigated the extent to which the Q-ASC (Ormond et al., 2018) discriminates between 5 and 12 year old autistic females and males compared to neurotypical females and males. The key hypothesis was that the seven Q-ASC subscales of Gendered Behaviour, Sensory Sensitivity, Compliant Behaviour, Friendships and Play, Social Masking, Imagination, and Imitation would discriminate between autistic females and nonautistic (neurotypical) females and would discriminate between autistic females and autistic males. We also explored the extent to which two subscales, Sensory Sensitivity and Compliant Behaviour, discriminated between autistic and neurotypical children.

Method.

- *Participants*

Participants were parents of 323 children aged 5 to 12 years (*M* = 8.06, *SD* = 2.25), 111 of whom had a diagnosis of ASD-Level 1, and 212 of whom did not have a clinical/ASD-Level 1 diagnosis (‘neurotypical’ children). Table 1 provides a gender and age breakdown for both the autistic sample and the neurotypical sample. Table 2 provides data on the primary and comorbid diagnoses of the autistic group.

**Table 1:** Ages split by diagnostic group and gender (N = 323)

	Autistic children		Neurotypical children	
Diagnostic Category	Males	Females	Males	Females
	64	47	68	144
Age *M(SD)*	8.79 (2.07)	8.86 (2.20)	7.72 (2.16)	7.51 (2.31)

**Table 2:** Primary diagnoses split by Gender and Autism Diagnosis

	Males (*n* = 64)		Females (*n* = 47)	
Clinical sample characteristics	n	%	n	%
Primary Diagnosis				
Asperger’s Syndrome (AS)*	31	48.4	30	63.8
Autism Spectrum Disorder (ASD)*	27	42.2	11	23.4
Autism (High Functioning*)	3	4.7	1	2.1
Pervasive Developmental Disorder Not Otherwise Specified (PDDNOS)	3	4.7	5	10.7
Co-occurring Diagnosis^a^				
Yes^b^	21	32.8	19	40.4
No	43	67.2	28	59.6

*Note*. ^a^*n* = 111, ^b^*n* = alternative diagnostic labels included: depression, Attention Deficit Hyperactivity Disorder (ADHD), Oppositional Defiant Disorder (ODD), Anxiety, Obsessive, Eating Disorder (Anorexia, Bulimia) Compulsive Disorder (OCD). * For the purposes of this table we retain the diagnosis reported by parents as having been given, and acknowledge that these diagnostic terms have been discarded

Archival clinical participant data were obtained from a specialist autism psychology clinic located in Brisbane, Australia. To meet eligibility for inclusion in the study, parents confirmed that their child had received a diagnosis of ASD-Level 1 (autism without language or intellectual impairment) and were in the selected age range (5–12 years). All archival data was de-identified to protect the privacy of all participants. Diagnostic protocol and conferment of the current sample was deemed clinically appropriate at the expert discretion of clinical psychologist diagnosticians. Neurotypical participants were recruited through social media (e.g., Facebook) and school newsletters. To meet eligibility for inclusion in this subgroup, parents confirmed that their child had not received a clinical diagnosis of autism or a neurodevelopmental disorder, did not attend a special school, and were in the selected age range (5–12 years). Furthermore, the non-clinical sample were screened using *The Autism Spectrum Screening Questionnaire - Girl* (ASSQ-GIRL; Kopp & Gillberg, 2011) for features consistent with the autistic female presentation clinically identified in both males and females to ensure integrity of the sample.

- *Measures*

The *Modified Questionnaire for Autism Spectrum Conditions***(**Q-ASC-M: Attwood et al., 2011; Ormond et al., 2018) is a 36-item questionnaire designed to assess parent/caregiver perceptions of behaviours and abilities associated with autism in children aged 5–19 years, including those pertaining to the female presentation of ASD-Level 1. Respondents are required to rate their level of agreement with each item on a four-point scale ranging from *1* ‘*definitely disagree*’ to *4* ‘*definitely agree’*. The subscales of the modified version have demonstrated adequate internal consistency: Gendered Behaviour (5 Items, α = 0.86; e.g., “Is s/he interested in looking feminine?); Sensory Sensitivity (6 Items, α = 0.72; e.g., “Is s/he bothered by bright lights or certain kind of lights?”); Compliant Behaviour (5 Items, α = 0.72; e.g., “Is s/he well-behaved at home?); Friendships & Play (5 Items, α = 0.76; e.g., “Does s/he enjoy playing with others?”); Social Masking (5 Items, α = 0.61; e.g., “Does s/he have a facial ‘mask’ that hides his/her social confusion?); Imagination (5 Items, α = .67; e.g., “Is s/he interested in fiction?”); and Imitation (5 Items, α = .62; e.g., “Does s/he copy or clone him/herself on others?”). Items on each subscale are summed to produce a total subscale score. With the exception of the sensory sensitivity subscale, subscale scores may range from 5–20, with higher scores indicating greater autism-consistent behaviours in the various areas. Scores on the Sensory Sensitivity subscale may range from 6–24, with higher scores indicating greater problems with sensory sensitivity.

The *Autism Spectrum Screening Questionnaire - Girls* (ASSQ-GIRL; Kopp & Gillberg, 2011) is an 18-item parent or caregiver-rated questionnaire designed to assess behavioural characteristics consistent with an emerging female presentation of autism in child and adolescent females. Parents/caregivers rate their level of agreement with each item on a three-point scale (*0* ‘No’, *1 ‘*Somewhat’, *2* ‘Yes’) across 18 items (α = 0.94; e.g., “Interacts mostly with younger children”). Total scores may range from 0 to 36, with higher scores indicating greater reported levels of features consistent with the female presentation of autism. A cut-off score of 20 indicates a greater likelihood of ASD-Level 1 (Kopp & Gillberg, 2011). Therefore, it was necessary for all non-clinical participants to score below 20 for inclusion in the study. The ASSQ-GIRL has demonstrated high internal consistency *(a* = 0.94) and good convergent validity (*r* = .85, *n* = 191; *p* < .001) for males and females with a clinical presentation.

- *Procedure*

Prior to commencement of the study, ethics approval was sought and granted by the Griffith University Human Research Ethics Committee. All archival data was de-identified prior to the transfer and safe storage of electronic and paper-based questionnaire responses for analysis. All non-clinical data was collected using secure online survey software. Participants accessed the questionnaire via a weblink and provided tacit informed consent of the research terms stated in the Information and Consent Form prior to completing the online survey. Participants confirmed their understanding of the purpose of the study, inclusion criteria, the expected time to complete the questionnaire (approximately 10 min), details of voluntary, confidential, and anonymous participation, and their ability to withdraw at any time without penalty. Only those who provided informed consent were able to proceed to the questionnaires. Incentive to participate in the current study was offered as inclusion in a prize draw to win an iPad or a range of $50 gift cards. Entering the draw was optional and was not linked to survey responses.

- *Analysis*

All statistical analyses were performed in IBM SPSS version 26. Power analyses conducted using G*Power (Faul et al., 2007), with a power of 0.80, a medium effect size (*OR* = 3.47) for logistic regression, and an alpha of 0.05, suggested that a total sample size of 101 participants were required for analyses. Thus, the current sample of 323 participants was sufficient.

To assess the ability of the Q-ASC to accurately discriminate between autistic and nonautistic (neurotypical) females and males, two hierarchal binary logistic regressions were used, one for females and one for males. For each regression analysis, age was controlled by entering this on Step 1 of modelling, then the 7 Q-ASC subscale scores were entered on Step 2. The Hosmer and Lemeshow Test was used to examine model fit, in addition to the Cox and Snell *R*^*2*^ to ascertain the amount of variance the subscale variables add over and above the other variables. The Wald test was used to ascertain the relative contribution of each independent variable for predicting the likelihood of group membership.

To examine differences between autistic females and males, seven univariate ANOVAs were conducted to examine the difference between gender, and autistic/neurotypical status. The dependant variables were scores on the seven subscales of the Q-ASC: Gendered Behaviour, Sensory Sensitivity, Compliant Behaviour, Friendships & Play, Social Masking, Imagination, and Imitation. The between groups variables were gender (female and male) and autistic/neurotypical status (autistic and nonautistic diagnosis). Therefore, these models include two main effects (gender and autistic/neurotypical status), and an interaction (gender X autistic/neurotypical status). Follow-up difference tests were conducted to examine any main effects and interactions.

Results.

- *Data Cleaning*

Prior to analyses, the data was cleaned to identify any outliers, data entry errors, and missing data (Tabachnick & Fidell, 2013). No univariate outliers were detected through examination of the standardized residuals, and undue influence was checked with Cook’s Distances and DFbeta values. Four female participants were found to have Cook’s Distance values outside acceptable limits (> 1) and were removed to avoid undue influence (Tabachnick & Fidell, 2013). Further, multicollinearity, logit linearity, and independence of errors were examined and found to be within acceptable limits (Field, 2014). The means and standard deviations of each of the seven subscales of the Q-ASC-M by age and gender are provided in Table 3.

**Table 3:** Descriptive variables and Q-ASC Subscales for females (N = 132) and males (N = 191)

		*Age*	*Gendered Behaviour*	*Sensory Sensitivity*	*Compliant Behaviour*	*Friendship and Play*	*Social Masking*	*Imagination*	*Imitation*
*FEMALES*									
*Autism group*	*Mean*	8.79	11.19	18.83	12.30	14.00	14.11	15.04	13.15
	*SD*	2.07	3.46	4.06	3.02	2.69	3.13	3.57	2.57
*Neurotypical group*	*Mean*	7.72	8.45	9.78	15.60	16.97	10.15	15.56	11.06
	*SD*	2.16	2.16	3.30	2.69	2.04	2.89	2.81	2.45
*MALES*									
*Autism group*	*Mean*	8.86	8.42	17.20	11.92	14.17	12.11	13.80	9.88
	*SD*	2.20	2.47	4.31	3.41	3.24	3.21	3.46	3.34
*Neurotypical group*	*Mean*	7.51	7.91	10.09	15.38	16.57	10.24	14.51	9.47
	*SD*	2.31	2.01	3.85	2.95	2.34	2.73	2.93	2.57

The binary logistic regression of autistic/neurotypical status on Q-ASC subscales and age was significant for females, χ^2^ (7) = 179.12, *p* < .001, Cox and Snell *R*^2^ = 0.62, Nagelkerke *R*^2^ = 0.93, explaining 92.8% of the variance in ASD-Level 1 diagnosis. The Hosmer and Lemeshow test confirmed that the model was a good fit for the data, χ^2^ (*df* = 8) = 3.84, *p* = .87. Coefficients for the model are presented in Table 4. As shown in Table 5^5^, age, Gendered behaviour, Sensory Sensitivity, Compliant Behaviour, Imagination, and Imitation were significant predictors in the final model. It was found that for every year increase in age, there was a 3.28 increase in the chance of an ASD-Level 1 diagnosis. Within the subscales, each unit increase in Gendered Behaviour, Sensory Sensitivity, Imagination, and Imitation there was a 2.86, 3.28, 3.18, 2.11, and 3.52 times increase in the chance of an ASD-Level 1 diagnosis, respectively. Additionally, with each unit increase in compliant behaviour, there was a 57.7% reduction in the likelihood of an autism diagnosis.

**Table 4:** Hierarchical Binary Logistic Regression of autistic/neurotypical status for female children (N = 191)

	*b*	*SE (b)*	*Exp(B)*	*95% CI*
*Step 1*				
Constant	-3.23*	0.70	0.05	
Age	0.25*	0.08	1.28	1.09–1.51
*Step 2*				
Constant	-45.13**	18.80	0.01	
Age	1.19**	0.55	3.28	1.12–9.55
Gendered Behaviour	1.05**	0.45	2.86	1.20–6.84
Sensory Sensitivity	1.16*	0.37	3.18	1.53–6.58
Compliant Behaviour	-0.86**	0.40	0.42	0.19–0.92
Friendships and Play	-0.86	0.49	0.42	0.16–1.10
Social Masking	0.38	0.23	1.46	0.93–2.28
Imagination	0.75*	0.35	2.11	1.07–4.17
Imitation	1.26*	0.48	3.52	1.37–9.05

*Note.* *p < .01, **p < .05

The binary logistic regression of autistic/neurotypical status on Q-ASC subscales and age was significant for males, χ^2^ (7) = 76.58, *p* < .001, Cox and Snell *R*^2^ = 0.49, Nagelkerke *R*^2^ = 0.65, explaining 64.7% of the variance in autism diagnosis (present/absent). The Hosmer and Lemeshow test confirmed that the model had good fit to the data (Hosmer-Lemeshow Goodness of fit χ^2^ (8) = 12.83, *p* = .12. Coefficients for the model are presented in Table 5. As shown in Table 5, in the final model, age, sensory sensitivity and compliant behaviour were predictive of autism diagnosis. It was found that for every increase of one year, there was a 1.29 timed increase in the chance of an autism diagnosis, for each unit increase in sensory sensitivity, there was a 1.36 increase in the chance of an ASD-Level diagnosis, and for each unit increase in compliant behaviour, there was a 23.4% reduction in the likelihood of an autism diagnosis.

**Table 5:** Exploratory analysis of autistic/neurotypical status for male children (N = 132) using hierarchical logistic regression

	*b*	*SE (b)*	*Exp(B)*	*95% CI*
*Step 1*				
Constant	-2.16*	0.68	0.05	
Age	0.26*	0.08	1.26	1.11–1.51
*Step 2*				
Constant	1.08	3.047	0.13	
Age	0.26***	0.12	1.29	1.02–1.64
Gendered Behaviour	-0.08	0.12	0.92	0.73–1.16
Sensory Sensitivity	0.30*	0.07	1.36	1.18–1.56
Compliant Behaviour	-0.27**	0.10	0.77	0.63–0.93
Friendships & Play	-0.11	0.12	0.89	0.71–1.12
Social Masking	0.09	0.11	1.09	0.89–1.35
Imagination	-0.09	0.09	0.91	0.76–1.09
Imitation	-0.09	0.10	0.91	0.76–1.10

*Note.* *p < .001, **p < .01, ***p < .05

The main effects and interactions from the ANOVAs conducted on the seven Q-ASC subscales can be seen in Table 6. The results of the follow-up difference tests can be seen in Table 7. The results showed statistically significant interactions between gender and autistic/neurotypical status for Gendered Behaviour, Sensory Sensitivity, Social Masking, and Imitation. It was found that females with an autistic diagnosis had statistically significantly higher scores on the Gendered Behaviour (M^Diff^ = 3.08), Sensory Sensitivity (M^Diff^ = 2.18), Social Masking (M^Diff^ = 2.28), and Imitation (M^Diff^ = 3.26), when compared to males with an autistic diagnosis. No differences were detected between males and females in the nonautistic group, with the exception of Sensory Sensitivity, where females scored significantly higher than males (M^Diff^ = 1.57).

**Table 6:** Main effects and interactions for the Q-SAC subscales across gender and autistic/neurotypical status (N = 323)

*Variable*	*F*	*df*	*np* ^*2*^
*Gendered Behaviour*			
Autism Diagnosis	36.81*	1,315	0.11
Gender	38.18*	1,315	0.11
Autism Diagnosis * Gender	18.72*	1,315	0.06
*Sensory Sensitivity*			
Autism Diagnosis	353.04*	1,315	0.53
Gender	4.17***	1,315	0.01
Autism Diagnosis * Gender	8.12**	1,315	0.03
*Compliant Behaviour*			
Autism Diagnosis	91.15*	1,315	0.22
Gender	0.48	1,315	0.00
Autism Diagnosis * Gender	0.01	1,315	0.00
*Friendships & Play*			
Autism Diagnosis	84.31*	1,315	0.21
Gender	0.01	1,315	0.00
Autism Diagnosis * Gender	1.55	1,315	0.01
*Social Masking*			
Autism Diagnosis	72.53*	1,315	0.19
Gender	9.14**	1,315	0.03
Autism Diagnosis * Gender	10.94*	1,315	0.03
*Imagination*			
Autism Diagnosis	2.82	1,315	0.01
Gender	8.44**	1,315	0.03
Autism Diagnosis * Gender	0.05	1,315	0.00
*Imitation*			
Autism Diagnosis	14.53*	1,315	0.04
Gender	54.33*	1,315	0.15
Autism Diagnosis * Gender	6.64**	1,315	0.02

Note: *p < .001, **p < .01, ***p < .05

**Table 7:** Follow-up difference tests for the Q-SAC subscales across gender and autistic/neurotypical status (N = 323)

*Variable*	*M* ^*Diff*^	*SE*	*CI (95%)*
*Gendered Behaviour*			
Autism Diagnosis (NoADx vs. ADx)	-1.78*	0.29	-2.35, -1.20
Gender (F vs. M)	1.81*	0.29	1.23, 2.39
Autism Diagnosis * Gender (NoADx: F vs. M)	0.54	0.35	-0.15, 1.24
Autism Diagnosis * Gender (ADx: F vs. M)	3.08*	0.47	2.16, 4.00
*Sensory Sensitivity*			
Autism Diagnosis (NoADx vs. ADx)	-8.39*	0.45	-9.27, -7.51
Gender (F vs. M)	0.91***	0.45	0.33, 1.79
Autism Diagnosis * Gender (NoADx: F vs. M)	-0.36	0.54	-1.42, 0.70
Autism Diagnosis * Gender (ADx: F vs. M)	2.18**	0.71	0.78, 3.59
*Compliant Behaviour*			
Autism Diagnosis (NoADx vs. ADx)	-3.45*	0.36	-4.16, -2.74
Gender (F vs. M)	0.25	0.36	-0.46, 0.96
Autism Diagnosis * Gender (NoADx: F vs. M)	0.24	0.44	-0.62, 1.10
Autism Diagnosis * Gender (ADx: F vs. M)	0.26	0.58	-0.88, 1.40
*Friendships & Play*			
Autism Diagnosis (NoADx vs. ADx)	-2.78*	0.30	-3.37, -2.18
Gender (F vs. M)	0.22	0.30	-0.57, 0.62
Autism Diagnosis * Gender (NoADx: F vs. M)	0.40	0.36	-0.32, 1.11
Autism Diagnosis * Gender (ADx: F vs. M)	-0.35	0.48	-1.31, 0.60
*Social Masking*			
Autism Diagnosis (NoADx vs. ADx)	-3.06*	0.36	-3.77, -2.36
Gender (F vs. M)	1.09**	0.36	0.38, 1.80
Autism Diagnosis * Gender (NoADx: F vs. M)	-0.10	0.43	-0.95, 0.75
Autism Diagnosis * Gender (ADx: F vs. M)	2.28*	0.58	1.15, 3.41
*Imagination*			
Autism Diagnosis (NoADx vs. ADx)	-0.63	0.38	-0.11, 1.37
Gender (F vs. M)	1.10**	0.38	0.35, 1.84
Autism Diagnosis * Gender (NoADx: F vs. M)	1.01***	0.45	0.12, 1.90
Autism Diagnosis * Gender (ADx: F vs. M)	1.18	0.60	-0.01, 2.37
*Imitation*			
Autism Diagnosis (NoADx vs. ADx)	-1.25*	0.33	-1.89, -0.60
Gender (F vs. M)	2.42*	0.33	1.77, 3.06
Autism Diagnosis * Gender (NoADx: F vs. M)	1.57*	0.39	0.80. 2.35
Autism Diagnosis * Gender (ADx: F vs. M)	3.26*	0.52	2.23, 4.29

*Note*. *p < .001, **p < .01, ***p < .05, F = female, M = male, ADx = autistic diagnosis, NoADx = No autistic diagnosis

Discussion

There is increasing acceptance and clinical recognition that autism may present differently in female children compared to male children (Attwood, 2012; Chawarska et al., 2016; Cridland et al., 2014; Dworzynski et al., 2012; Garnett et al., 2013; Wilkinson, 2008). Systematic ways to identify the apparently subtle and complex characteristics in autistic females are yet to be established, yet the need for clinicians and diagnosticians to be able to identify autism accurately and sensitively in both genders is paramount. This study aimed to investigate the ability of the Q-ASC-M, to accurately discriminate between autistic and nonautistic (neurotypical) females and males aged 5–12 years.

The results were generally consistent with the key hypothesis. Compared to autistic males, autistic female children had higher scores on gendered behaviour, sensory sensitivity, social masking, and imitation. Gender differences between autistic children on compliant behaviour, friendships and play, and imagination were not significant. Gendered behaviour, sensory sensitivity, compliant behaviour, imagination, and imitation were all found to discriminate between autistic females compared to neurotypical females. The findings that social masking and friendship and play did not discriminate between neurotypical and autistic females were inconsistent with the key hypothesis. Exploratory analyses indicated that the sensory sensitivity and compliant behaviour subscales discriminated between autistic and neurotypical male children.

Both gender differences and differences across diagnostic categories on Q-ASC scales indicate that autistic females present differently from their male counterparts and neurotypical females. In particular, the results for gendered behaviour are novel as there has been little empirical research conducted on gender typicality in autistic females previously, and are consistent with clinical observations that autistic females often present as gender-atypical or may actively defy social gender conventions (Faherty, 2006). They also align with the results of Ormond et al. (2018) who found that parents reported a greater level of observed incongruence in gendered behaviour for autistic females than autistic males. Similarly, the findings for imitation are consistent with research and clinical observation suggesting that autistic females may undertake a cognitive process of imitation as a social-cognitive defence due to social and communication deficits, awareness of identity, and sense of self (Giarelli et al., 2010; Goddard et al., 2014). This may appear as avidly observing others socially, adopting a different persona, or copying or cloning someone identified as socially successful as a potential mask for social deficits (Glidden et al., 2016). Finally, the results for imagination are consistent with Kopp and Gillberg (2011) who found that females were more likely to engage in fantasy and fiction, which were associated with behavioural characteristics that served as a function to ease social anxiety.

Contrary to the key hypothesis, the friendships and play and social masking subscales did not discriminate between autistic and neurotypical female children, suggesting that these two subscales may not be useful in their current form when screening for autism in females. It may be that these characteristics are more evident in adolescents rather than younger children. Indeed, Ormond et al. (2018) found that parents of adolescents reported lower levels of friendships and play characteristics than parents of younger children, suggesting a subtler presentation in earlier developmental years. This may also be the case with social masking, with this skill perhaps requiring greater cognitive ability and maturity. Future research should replicate this study with adolescents to assess whether differences in friendships and play and social masking are evident between those with and without autism in an older cohort.

The domains of sensory sensitivity and compliant behaviour significantly discriminated between children with and without autism for both genders, which demonstrate congruence of these two characteristics across genders. Thus, compared to parents of neurotypical children, parents of autistic children report sensory sensitivities and noncompliance (i.e., greater behavioural difficulties, less compliance with requests, and disproportionate reactivity) in their children. It is interesting to note that previous clinical research suggests a variance in presentations across contexts, with greater externalising behaviours seen in autistic females at home, compared to school (Bulhak-Paterson, 2015; Willey, 2015). Indeed, autistic female children demonstrate an ability for social learning and present with compliant, helpful and socially acceptable behaviour at school as a learned approach for greater likability, and to camouflage their deficits, with no teacher-reported problems (Cridland et al., 2014). However, the resultant emotional suppression may present as a significant shift in behaviour at home, where children may display depressed mood, social confusion, and distress (e.g., externalised behaviour or meltdowns; Attwood, 2007; Gould & Ashton-Smith, 2011). Thus, noncompliance in autistic female children may well be picked up by parents but not teachers, highlighting the importance of parent report in the diagnosis of autism in females.

Autistic females who remain undiagnosed, or who are incorrectly diagnosed with and treated for alternative psychopathology, may experience significant and detrimental economic and health impacts in later life as a result (Wilkinson, 2008). The benefit of early and accurate diagnosis has been shown to align with appropriate and effective treatments that contribute to better management and adaptation to symptoms and provide a sense of meaning and greater levels of wellbeing (Giarelli et al., 2010; Gould & Ashton-Smith, 2011). The results of this study suggest that the Q-ASC-M screens well for females with AS.

This study is limited by its cross-sectional design, so causality cannot be established. The neurotypical sample was screened for autism using the ASSQ-GIRL and cut-off scores relating to the female presentation of autism (Kopp & Gillberg, 2011), but they were not assessed by a trained clinician. Future research should ensure that control groups are assessed by trained professionals to ensure that they are indeed neurotypical. Because limited demographic information was available, it is unclear how generalisable these results are across groups varying in socioeconomic status and other key demographics.

- Conclusions

The results of this study suggest that the Q-ASC-M is adequate in its ability to discriminate between autistic males and females and nonautistic (neurotypical) individuals, with greater discriminatory capacity for females compared than males. Only two of the seven subscales discriminated between males with and without AS, whereas five of the seven subscales discriminated between females with and without AS. Q-ASC scores on gendered behaviour, sensory sensitivity, social masking, and imitation may assist health and education professionals in detection of autism in female children.
